# Study of retina and choroid biological parameters of rhesus monkeys eyes on scleral collagen cross-linking by riboflavin and ultraviolet A

**DOI:** 10.1371/journal.pone.0192718

**Published:** 2018-02-08

**Authors:** Mingshen Sun, Fengju Zhang, Bowen Ouyang, Mengmeng Wang, Yu Li, Xuan Jiao, Li Zhang, Ningli Wang

**Affiliations:** 1 Beijing Tongren Eye Center, Beijing Tongren Hospital, Capital Medical University, Beijing Ophthalmology & Visual Sciences Key Lab, Beijing, China; 2 Hebei Ophthalmology Key Lab, Hebeisheng Eye Hospital, Xingtai, Hebei Province, China; University of Hong Kong, HONG KONG

## Abstract

To evaluate ocular fundus biological changes after scleral collagen cross-linking (CXL) with riboflavin/ ultraviolet A (UVA) on rhesus monkeys in vivo by analyzing retina and choroid biological parameters. Six 3-year-old male rhesus monkeys (12 eyes) were observed in this study, with scleral CXL procedures applied on superior temporal equatorial sclera on random eyes of all rhesus. Optical coherence tomography (OCT) and optical coherence tomography angiography (OCTA) examination were performed before and 1 week, 1 month, 3 months and 6 months after CXL. The thickness of retina and choroid and the flow density of retinal superficial vascular networks were analyzed respectively in different regions after CXL. As for retina thickness and flow density of retinal superficial vascular networks, no statistical difference was noted between CXL eyes and control eyes at 1 day, 1week, 1 month, 3 months and 6 months (P>0.05). Among choroid parameters, the choroidal thickness in 1500μm temporal to the fovea center of CXL eyes revealed a significant reduction in 1 week postoperatively (P<0.05), but it subsequently increased from 1 month postoperatively, and no statistical difference was found between two groups in the following periods (P>0.05). The choroidal thickness nearby crosslinked region may change temporarily following scleral CXL, and it might recover gradually after 1 month postoperatively. The vascular flow density and thickness of retina were not affected by scleral CXL. Further study should be performed to evaluate the potential adverse effects at the direct vicinity of the application site and the long-term effect of scleral CXL in clinical application.

## Introduction

Myopia is one of the most common ocular disorder and has become the leading cause of visual impairment and blindness in Asian countries [1–2]. The prevalence of high myopia among young adults in Asia was reported to be 6.8–21.6%, which was higher than non-Asian population (2.0%-2.3%) [2]. High myopia, especially pathological myopia, is associated with an excessive axial elongation of eyeball, which probably caused by scleral biomechanical weakness and thinning [3]. Until now, many therapeutic attempts to resist myopic progression have been developed, including various posterior scleral reinforcement surgery [4–6]. Nevertheless, the efficient of these methods is controversial, and no evidence shows that the internal structure of the weakened sclera can be regenerated by these means.

Since corneal cross-linking (CXL) using riboflavin and ultraviolet A (UVA) became an increasingly common therapy for stabilizing the cornea and preventing keratoconus progression [7–9], this technique has also been suggested and investigated as an approach to stiffen the biomechanically weakened scleral tissue [10–11]. However, the safety of scleral CXL is still disputed. It was found that scleral CXL with riboflavin and UVA irradiation with an intensity of 4.2mW/cm^2^ for 30 min could lead to serious side effects in retinal tissue [12]. When the effective dose of UVA irradiation down to 3mW/cm^2^ for 30 min, there were no structural damage in retina of cross-linked rabbit eyes [13]. Recently, it was demonstrated that scleral CXL with riboflavin and UVA irradiation (57mW/cm^2^ for 200s) effectively prevents occlusion-induced axial elongation in a rabbit model [14]. The exploration of iontophoresis-assisted accelerated riboflavin/UVA scleral CXL also suggested that the modified scleral CXL procedure may be a potential method to control the pathologic process of myopia [15].

However, previous researches mainly focused on changes of scleral biomechanical properties and histopathological evaluation after scleral CXL to estimate the efficacy and safety of this treatment. Some literature also investigated changes of intraocular pressure (IOP) on scleral reinforced eyes, since it was known that IOP is closely associated with myopia [16–17]. But the results were still controversial in different scleral reinforcement methods [4, 18, 19]. Studies from our research group investigated electroretinography (ERG) changes on non-primate animal models and indicated that the dark-adapted ERG amplitudes were statistically reduced 1 week, 1 month and 3 months postoperatively [20]. Other researches focused on ocular refraction and axial length before and after the treatment, and demonstrated that less myopia and axial elongation was observed in CXL eyes [14, 21]. Nevertheless, the observation of optical biological parameters in vivo scleral cross-linked eyes of primates were not included into these articles. Therefore, this present study was conducted in rhesus monkeys to investigate biological parameters of retina and choroid after scleral CXL, aiming to evaluate the biological changes and safety of scleral CXL by riboflavin/UVA on primates in vivo. Animal protocols were reviewed and approved by the Institutional Animal Care and Use Committee of Capital Medical University.

## Materials and methods

### Subjects

The experimental study included 6 male Rhesus macaque monkeys (Macaca mulatta) with a mean age of 3.0±0.4 years (range 2.8–3.5 years) and a mean weight of 4.9±0.5 kg (range 3.6–6.2 kg). All rearing and experimental procedures were reviewed and approved by the Institutional Animal Care and Use Committee of Capital Medical University (AEEI-2014-127) and were in compliance with the Association for Research in Vision and Ophthalmology Statement for the use of animals in ophthalmic and vision research. All the animals used in this study were housed in groups of between 10 and 25, with windows allowing the animals to view the outside. Animals were in a 4.3 square foot cage measuring 24 (L) x 27 (D) x 32 (H). Cages were made of stainless steel. The temperature of the housing room was maintained at 25–27°C and the relative humidity was 45~55%. Regular changes of equipment and toys were provided. Wood chip substrate was on the floor to provide opportunity for foraging. Foods were provided three times a day and water was available ad libitum. Facilities were in the rom to allow swinging and jumping and engaging in activities from floor to ceiling. Monkeys were health checked daily throughout the experiment. Before recruiting into the experiment, all rhesus monkeys were given comprehensive ocular examination, including anterior segment and fundus examination, to exclude any ocular disease. Rhesus monkeys were not sacrificed in order to be followed up for a long term observation.

Both eyes of the monkey were examined clinically before, and 1 week, 1 month, 3 months and 6 months after the scleral CXL operation. For all examinations and surgeries, the animals were anesthetized with an intramuscular injection of ketamine hydrochloride (20 mg/kg body weight; ketamine 5%) and xylazine hydrochloride (0.2 mg/kg body weight), with repeated injections of ketamine (10 mg/kg) as needed during the examination. While the measurements were being taken, the eyelids were gently held apart by eye speculum and the tear film was maintained by the frequent application of artificial tears (Sodium Hyaluronate 0.1%). Cycloplegia was achieved by topically instilling 1–2 drops of 5% tropicamide (Tropicamide phenylephrine eye drops) at 5 min interval 30 min before performing retinoscope, OCT and OCTA.

### Surgery and scleral CXL treatment

According to our previous scleral CXL laboratory technique [11, 20], each rhesus monkeys were randomly selected one eye for CXL procedure, and the contralateral eye was served as intra-individual controls. Animals were anesthetized by the above mentioned method. The cornea was anesthetized with 1–2 drops of 0.5% proparacaine hydrochloride before surgery. A lid speculum was placed into the fornix. The conjunctiva was incised in upper anterior quadrant of the CXL eye with a pair of scissors. Sutures (Dacron 5–0) were placed in treatment quadrant for towing extraocular muscles and holding the eyeball to allow better exposition of superior temporal equatorial sclera as the corss-linked area.

Photosensitizer solution containing 0.1% riboflavin (0.1% Riboflavin, 20% dextran 500, Peschke D, PESCHKE Trade, Huenenberg, Switzerland) was dropped minutely onto the irradiation zone 20 min before and during the 30 min irradiation period. UVA irradiation (365 nm) was applied perpendicular to the sclera of CXL eyes by a UVA device (UV-X 1000, Avedro, Waltham, America) with a surface irradiance of 3.0 mW/cm^2^ for 30 min (total dose of UVA, 5.4 J/cm^2^) at a distance of 5 cm from the sclera. The control eyes were exposed and dropped by photosensitizer solution without UVA irradiation. After surgery, the sutures around muscles were removed, and the conjunctiva was closed using a vicryl 7–0 thread (Ethicon Inc, Livingston, Scotland, UK). The animals were monitored till awakening and kept in cages. 0.3% Gatifloxacin eye gel was applied after surgery and four times a day in 1 week postoperatively to avoid infection.

### Ocular biometry

The intraocular pressure (IOP) was measured using the TonoVet™ rebound tonometer (Icare Finland Oy, Helsinki, Finland) at about 9 AM. According to the manufacturer’s recommended procedures, the equipment was programmed to average the IOP values of six consecutive, acceptable measurements and produce a reading of the mean IOP. Five readings (each a mean of six measurements; a total of 30 separate measurements) were obtained per eye, and the mean IOP value was calculated and reported. Each animal was positioned in a seated posture in the process of measuring IOP.

The refractive status of each eye, including spherical and cylindrical components, was measured by an experienced investigator using streak retinoscope (YZ24, 66 Vision Tech Co., Ltd, Suzhou, China) and recorded. Refractive errors were reported as the spherical equivalent (SE) in diopters (D).

The axial length of each eye was measured by A-scan ultrasonography implemented with a 10-MHz transducer (ODM-1000A, MEDA Co., Ltd. Tianjin, China). Rhesus monkeys were in the supine position with the head immobilized by a head holder. The device was programmed to average the values of eight acceptable measurements and produce a reading of the mean. Three readings were recorded and averaged.

### SD-OCT scan acquisition

Both eyes of each rhesus monkeys were scanned using the Spectralis SD-OCT device (software version 1.9.10.0; Heidelberg Engineering, Heidelberg, Germany). The scan was performed on ART 30 frames including 768 A scans. The automatic eye tracking technology maintains fixation on the retina. Only well-centered images with a signal strength of >20db were used for analysis.

Macular and retinal layers thickness were reported in an Early Treatment of Diabetic Retinopathy Study macular map (ETDRS), and the 1, 3 and 6 mm rings were considered for the analysis. The 1 mm ring was defined as central thickness. The intermediate and outer rings were divided into four zones designated as superior, nasal, inferior, and temporal. The numerical values recorded for each of the nine zones were used in the analysis of retina thickness.

With enhanced depth imaging (EDI) and manual caliper provided by the software, choroidal thickness (CT) values were measured from the outer border of the hyper-reflective RPE to the inner aspect of the sclera at seven locations: beneath the foveal center and at 500 -μm intervals up to 1500μm temporal and nasal to the fovea center.

All images were taken by the same trained examiner and all measurements were repeated 3 times at each point.

### OCTA scan acquisition

Both eyes of each rhesus monkeys were scanned using the RTVue XR with AngioVue (software version 2015.100.0.3; Optovue, Inc., Fremont, CA, USA), with a light source centered at 840 nm, a bandwidth of 50 nm, and an A-scan rate of 70,000 scans per second. Scans of macular areas were acquired and the scan size was 6.0 mm × 6.0 mm.

Flow density of retinal superficial vascular networks was calculated automatically using the above software and was defined as the average de-correlation value as previously described [22–23]. Five zones divided by 1 and 3 mm ETDRS rings were considered for the analysis.

All images were taken by the same trained examiner and all measurements were repeated 3 times at each point.

### Statistical analysis

Statistical analysis was performed using SPSS for Windows, version 19.0 (IBM-SPSS, Chicago, Illinois, USA). The measurements were presented as mean ± standard deviation (SD). Paired t-tests were used to evaluate ocular biological parameters differences between cross-linked and control eyes in different pre-/post-operative periods. In all tests, statistical significance was defined at a level of P < 0.05.

## Results

A total of 12 eyes of 6 male rhesus monkeys were enrolled into this study, 6 eyes in scleral CXL group and 6 eyes in control group. At the initial measurement, there were no statistically significant differences in the thickness of retina and choroid and the flow density of retinal superficial vascular networks between the two groups (each P>0.05).

No signs of inflammation were observed after scleral CXL and throughout the follow-up period. After surgery, all rhesus monkeys had normal corneas, anterior chambers and clear lenses. No vitreous or retinal lesion was observed clinically.

**Fig 1:**
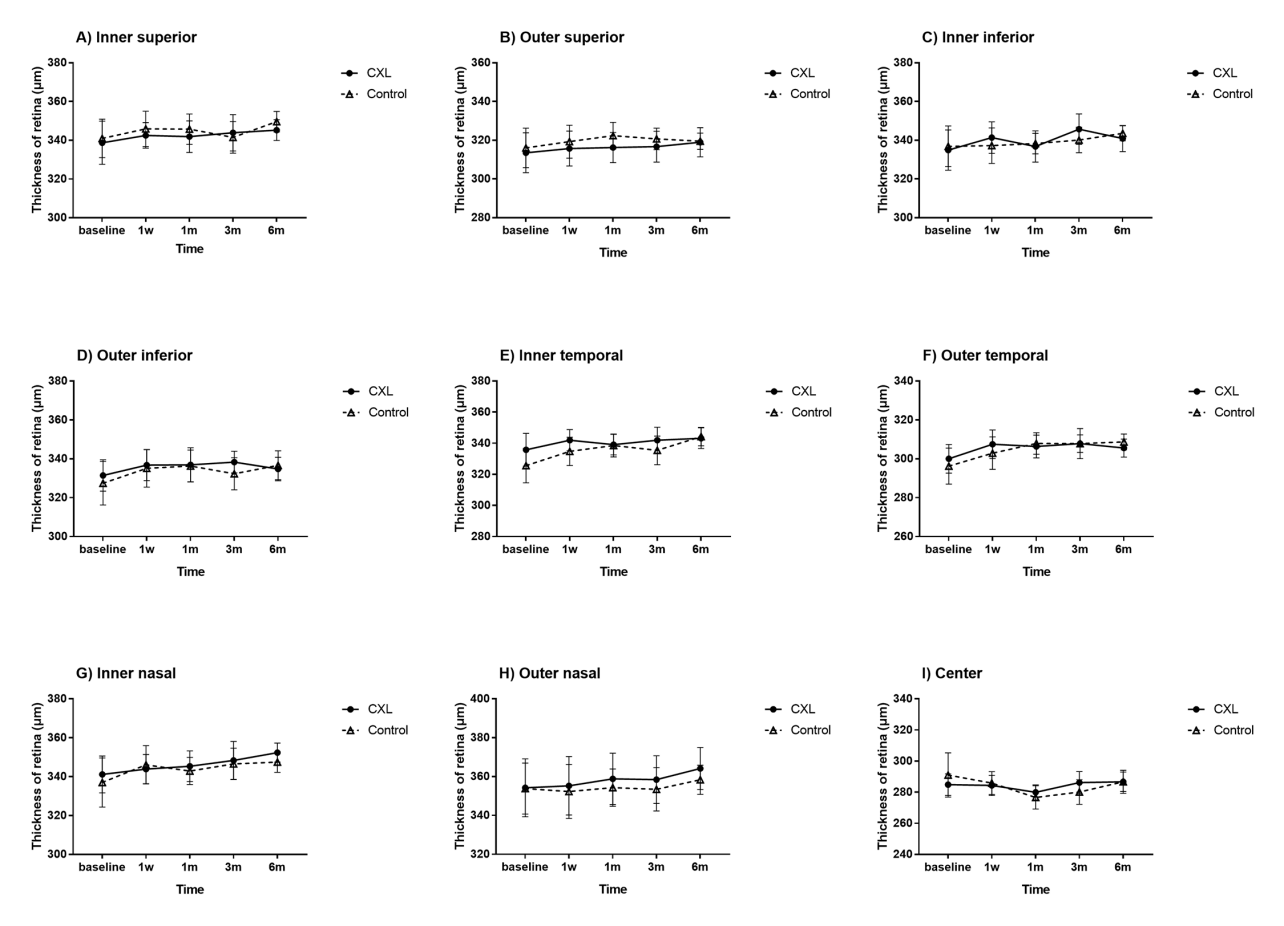
Retinal thickness of rhesus monkeys in different pre- and postoperative periods in scleral CXL group and control group. No statistical difference was noted between CXL eyes and control eyes at 1 day, 1 week, 1 month, 3 months and 6 months (each P>0.05) in these parameters. Error bar = 1 SEM.

**Fig 2:**
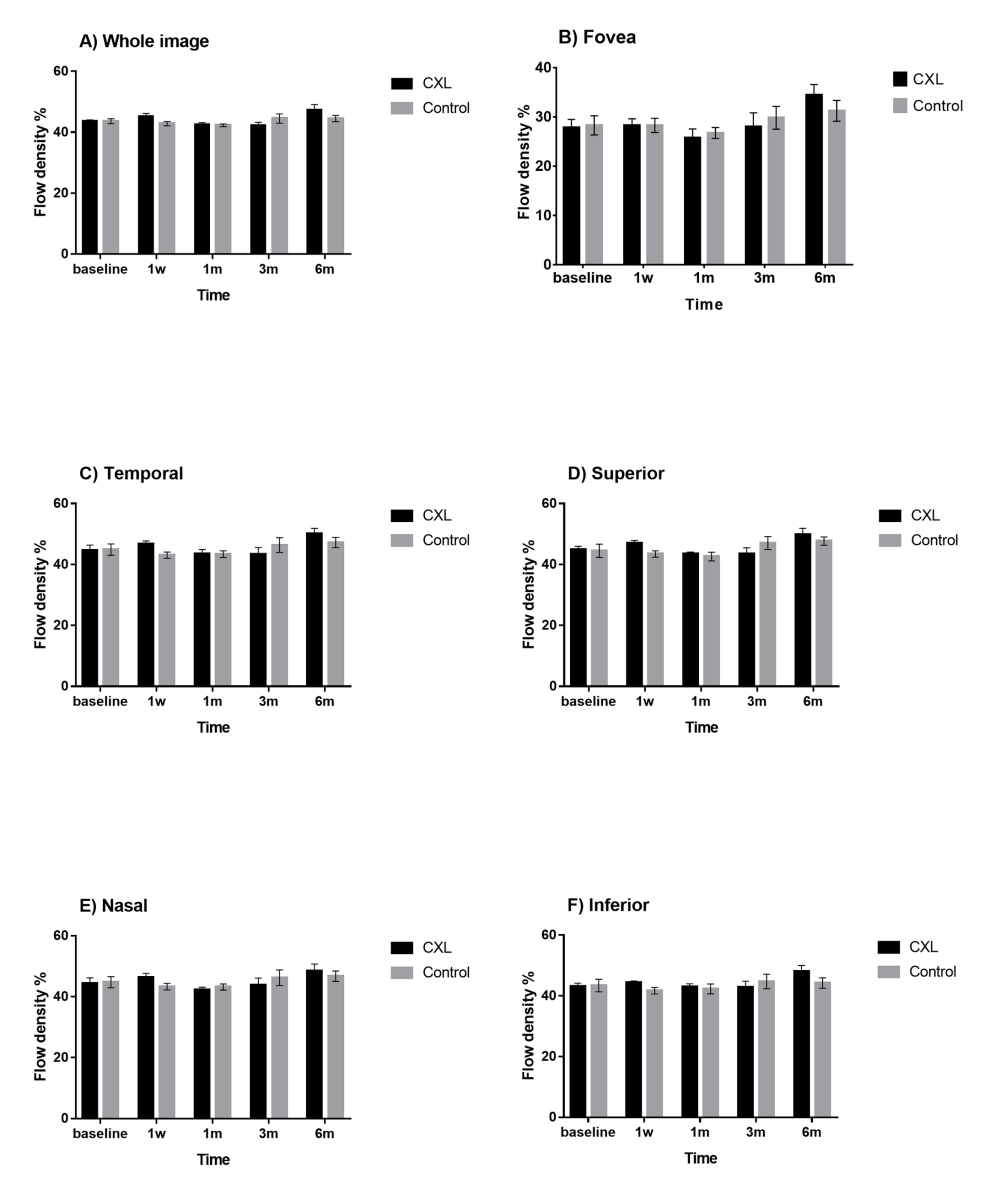
Flow density of retinal superficial vascular networks of rhesus monkeys in different pre- and postoperative periods in scleral CXL group and control group. No statistical difference was noted between CXL eyes and control eyes at 1 day, 1 week, 1 month, 3 months and 6 months (each P>0.05) in these parameters. Error bar = 1 SEM.

**Fig 3:**
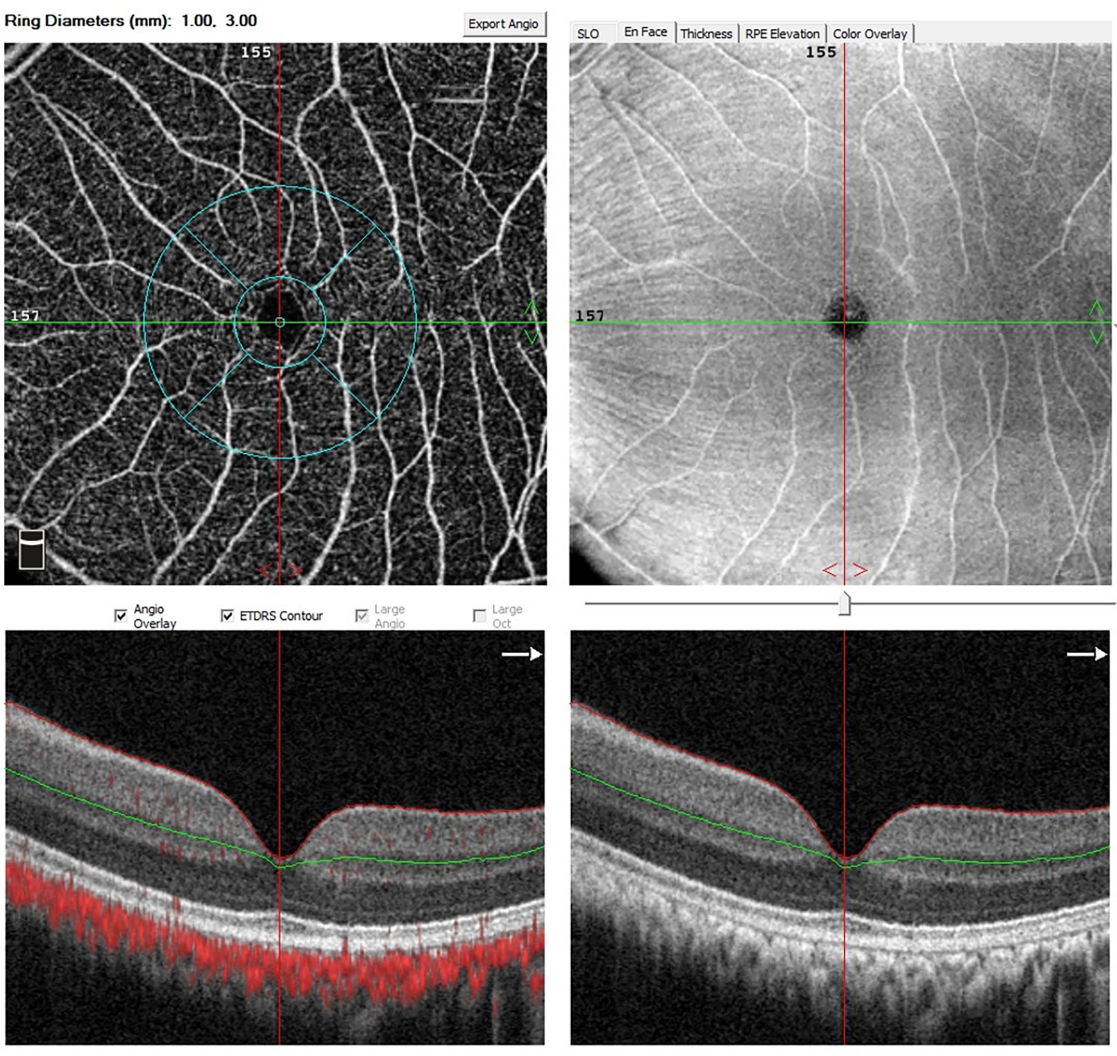
Optical coherence tomography angiographic image in CXL eye. Flow density of retinal superficial vascular networks was calculated automatically in ETDRS rings for the analysis.

**Fig 4:**
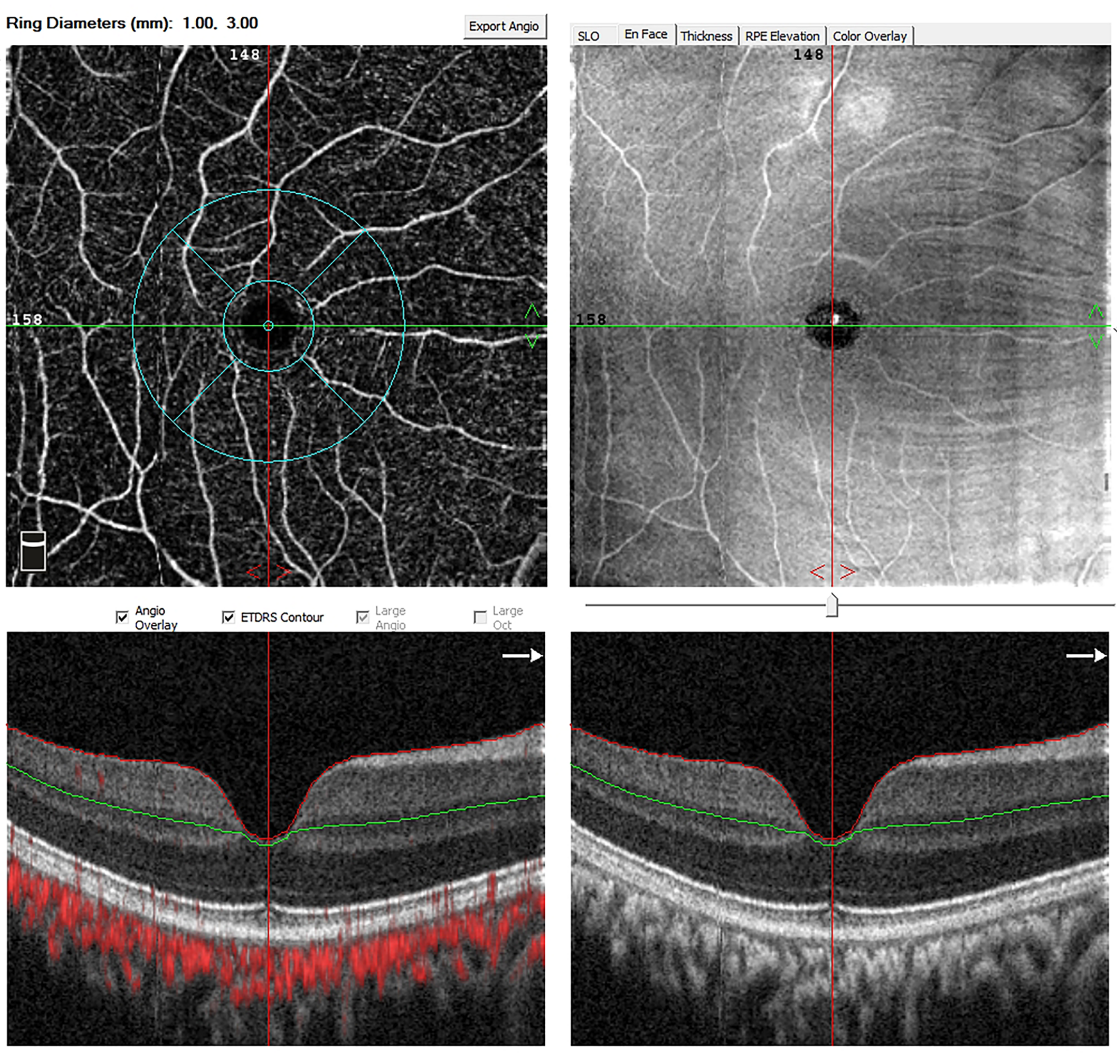
Optical coherence tomography angiographic image in control eye. Flow density of retinal superficial vascular networks was calculated automatically in ETDRS rings for the analysis.

Table 1 summarize IOP, spherical equivalent and axial length in different pre-/post-operative periods. The retinal thickness and flow density of retinal superficial vascular networks were summarized in Figs 1 and 2. The optical coherence tomography angiographic images in CXL eye and control eye were shown in Figs 3 and 4. No statistical difference was noted between CXL eyes and control eyes at 1 day, 1 week, 1 month, 3 months and 6 months (each P>0.05) in these parameters.

**Table 1:** IOP, spherical equivalent and axial length of rhesus monkeys in different pre- and postoperative periods in scleral CXL group and control group.

	IOP (mmHg)				Spherical equivalent (D)				Axial length (mm)			
	CXL	Control	t	p	CXL	Control	t	p	CXL	Control	t	p
**Baseline (n = 6)**	17.00±3.74	18.17±2.53	-1.202	0.283	1.20±1.16	0.70±1.11	0.877	0.430	19.63±0.62	19.55±0.63	1.305	0.249
**1 week (n = 6)**	17.70±2.38	19.57±2.60	-2.412	0.610	1.22±1.39	0.73±1.54	0.291	0.786	19.71±0.73	19.59±0.77	1.382	0.226
**1 month (n = 6)**	18.80±2.83	17.67±2.25	1.048	0.343	0.95±1.46	0.50±1.73	1.095	0.335	19.80±0.56	19.84±0.66	-0.591	0.580
**3 months (n = 6)**	18.60±4.00	18.33±4.33	0.437	0.680	0.83±1.37	0.25±1.59	0.650	0.330	19.83±0.52	19.83±0.72	0.069	0.947
**6 months (n = 6)**	21.38±2.13	20.70±1.22	0.579	0.588	0.65±0.22	0.25±0.56	1.138	0.100	19.87±0.51	19.89±0.63	-0.747	0.489

No statistical difference was noted between CXL eyes and control eyes at 1 day, 1 week, 1 month, 3 months and 6 months (each P>0.05) in these parameters.

**Fig 5:**
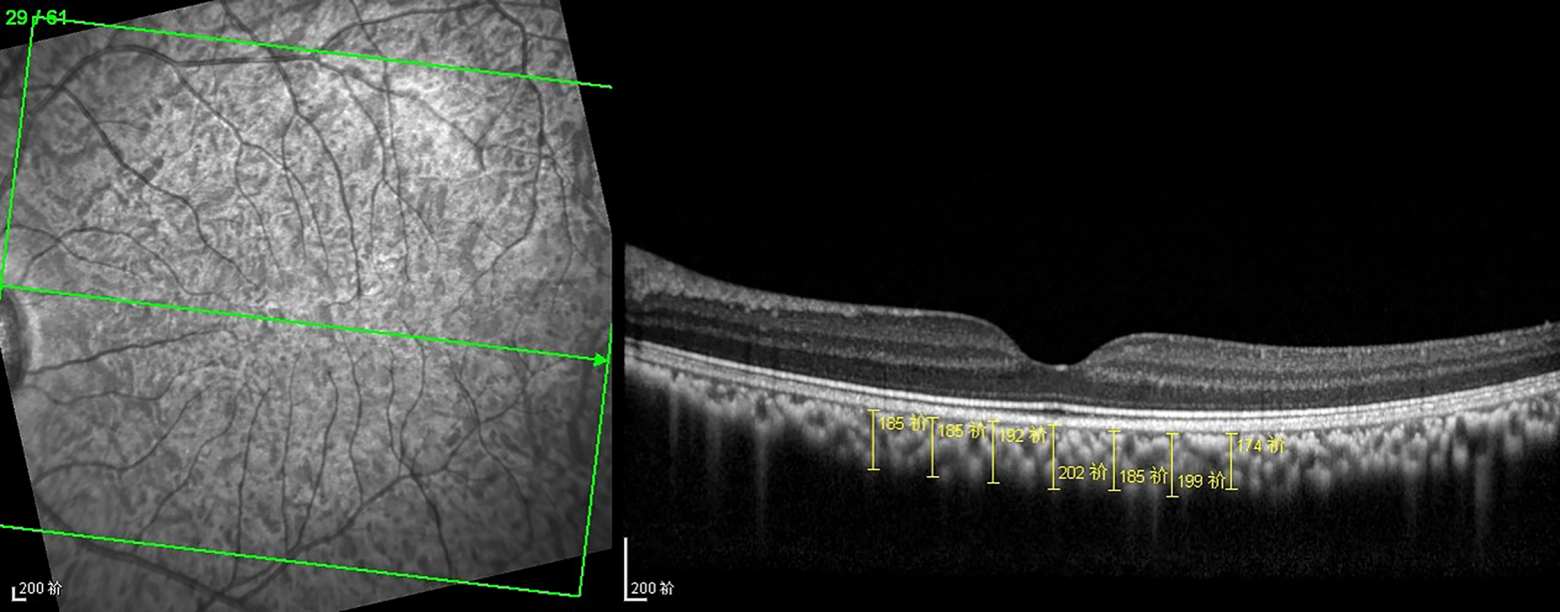
Optical coherence tomographic image in CXL eye. Choroidal thickness values were measured from the outer border of the hyper-reflective RPE to the inner aspect of the sclera at seven locations for the analysis.

**Fig 6:**
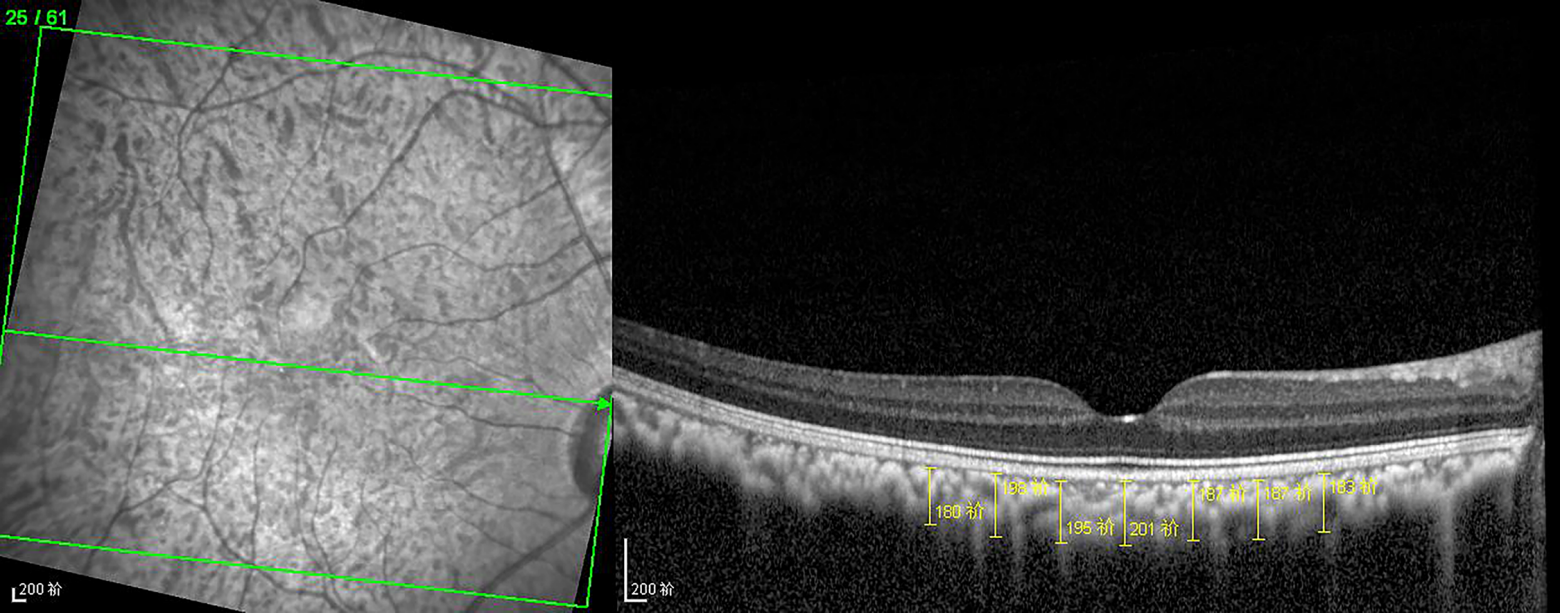
Optical coherence tomographic image in control eye. Choroidal thickness values were measured from the outer border of the hyper-reflective RPE to the inner aspect of the sclera at seven locations for the analysis.

**Fig 7:**
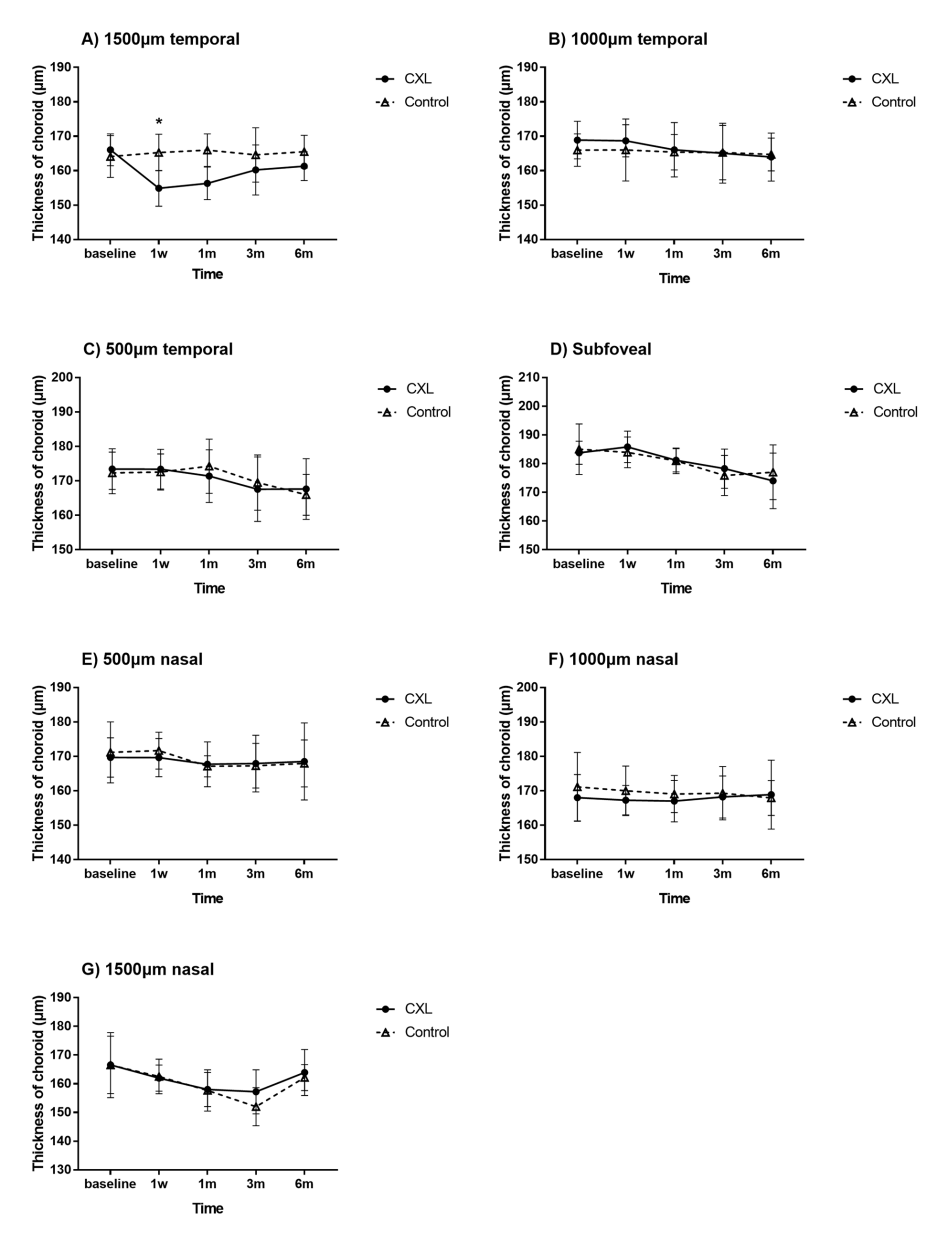
Choroidal thickness of rhesus monkeys in different pre- and postoperative periods in scleral CXL group and control group. The choroidal thickness in 1500μm temporal to the fovea center of CXL eyes revealed a significant reduction in 1 week postoperatively (P = 0.021). And there was no statistical difference in other zones between two groups (each P>0.05). The thickness of CXL eyes subsequently increase from 1 month postoperatively, and no statistical difference was noted between two groups in 1 month, 3 months and 6 months (each P>0.05). Error bar = 1 SEM. *p < 0.05.

The optical coherence tomographic images in CXL eye and control eye were shown in Figs 5 and 6. The choroidal thickness in 1500μm temporal to the fovea center of CXL eyes and control eyes were (154.88±12.68μm) and (165.29±13.05μm), which revealed a significant reduction in 1 week postoperatively in CXL eyes (P = 0.021). And there was no statistical difference in other zones between two groups (each P>0.05). The thickness of CXL eyes subsequently increase from 1 month postoperatively, and no statistical difference was noted between two groups in 1 month, 3 months and 6 months (each P>0.05) (Fig 7).

## Discussion

Controversy exists regarding the safety of scleral collagen cross-linking (CXL) by riboflavin/ultraviolet A (UVA), such as the ultraviolet-induced retinal damage. Compared with accelerated CXLs using shorter treatment times with higher UVA irradiations [24–25], the conventional CXL approach (3.0mW/cm^2^, 30 min) was applied in the present research for it has been generally studied and applied both in vivo experiments of scleral CXL [11, 13, 20] and in clinical practice of corneal CXL [26–27]. According to previous studies [11–13], the equatorial sclera was chosen as the treatment area of the scleral CXL procedures, and the scleral biomechanical strength was proved to be increased for more than 8 months postoperatively. While the safety results and the potential risk to the retina were still in doubt [28]. In this study, biological parameters of retina and choroid were investigated by SD-OCT and OCTA examinations, aiming to verify the safety of this scleral CXL technique in vivo primates.

In the analysis of retinal thickness, SD-OCT was performed pre- and postoperatively. There was no statistical difference between CXL eyes and control eyes at different pre-/post-operative periods in retinal thickness of the nine ETDRS subfields (each P>0.05). These outcomes indicated that the retinal thickness in measured area was not affected by scleral CXL in rhesus monkeys.

Flow density is the index reflecting quantitative measurement of vascular density [29]. In the present study, no statistical difference was noted between two groups at different pre-/post-operative periods in flow density of retinal superficial vascular networks (each P>0.05). It was demonstrated that the superficial vascular density in macular area was not affected by scleral CXL in rhesus monkeys.

The choroidal thickness in 1500μm temporal to the fovea center of CXL eyes revealed a significant reduction in 1 week postoperatively (P<0.05), but it subsequently increased from 1 month postoperatively, and no statistical difference was found between two groups in the following periods (each P>0.05). In other zones, there was no statistical difference between two groups postoperatively (each P>0.05). In previous studies [30–32], many factors could influence choroidal thickness, including age, axial length, sex and eye pressure, and it is still unknown how or whether these factors affect the local choroidal geometry. Hypothesis has been demonstrated that the choroidal circulation might play a role in the distribution of choroidal thickness [33–34]; thus, the decreased volume in the choroidal vascular bed would led to a reduction of choroidal thickness.

In this study, no statistical difference was noted in IOP, spherical equivalent and axial length between two groups in different pre-/post-operative periods. Thus, considering the sclera is a kind of tissue with sparse vasculature and penetrated by blood vessels supplying the choroid, we speculate that local capillaries within scleral tissue might be affected temporarily by CXL surgery, leading to reduced blood flow in irradiation zone. Therefore, the local choroidal circulation could be transiently impaired. It is possible that the decrease choroidal circulation caused thinner choroidal thickness in acute postoperative phase, and it may return to the normal level concomitant with the autonomic regulation [33, 35] subsequently according to outcomes in the present study. While the vascular flow density and thickness of retina were not affected by scleral CXL postoperatively.

Potential limitations of our study should be mentioned. First, retinal and choroidal parameters measured in vivo in this study is limited to posterior pole of eyeball, where is not underlying the treatment. However, there has not been any OCT equipment providing the software for measurement in equatorial region so far. Potential adverse effects at the direct vicinity of the application site should be investigated in further histological examination. Second, whereas manual choroidal thickness measurement is one of drawbacks of this study, until now, no automated measurement software is supplied by existing equipment. Finally, these results might be valid for rhesus monkeys, and it has not been clarified whether the results are applicable in other species.

In conclusion, the present study found that the choroidal thickness near crosslinked region may change temporarily following scleral CXL. This may be the result of reversible transient microcirculatory dysfunction of the choroid, and it might recover gradually after 1 month postoperatively. The vascular flow density and thickness of retina were not affected by scleral CXL. The potential role of scleral CXL in the effect of this change remain to be investigated. Further study should be performed to evaluate the pathological result and long-term effect of scleral CXL in clinical application.

## Supporting information

S1 Table Results of retina and choroid thickness and flow density of retinal superficial vascular networks in scleral CXL group and control group. (XLSX)
